# Twenty Years of Therapeutic Leukocytapheresis in Newly Diagnosed Acute Myeloid Leukemia: Insights From A Single Center

**DOI:** 10.1002/jca.70111

**Published:** 2026-03-16

**Authors:** Vojtech Latal, Ivana Skoumalova, Miroslava Palova, Tomas Szotkowski, Martin Cernan, Jana Navratilova, Helena Urbankova, Zuzana Pikalova, Ludek Raida, Edgar Faber, Tomas Papajik

**Affiliations:** ^1^ Department of Hemato‐Oncology, Faculty of Medicine and Dentistry Palacky University Olomouc and University Hospital Olomouc Olomouc Czech Republic

**Keywords:** acute myeloid leukemia, FLT3, hyperleukocytosis, KMT2A, leukocytapheresis, leukostasis, NPM1

## Abstract

Acute myeloid leukemia (AML) is a heterogeneous hematologic malignancy, and 5%–20% of newly diagnosed patients present with hyperleukocytosis (HL). HL, most often defined as WBC > 100 000/μL, is a hematologic emergency associated with severe complications, early mortality, and poor prognosis, requiring immediate intervention. From November 2005 to September 2025, 65 newly diagnosed AML patients with HL underwent leukocytapheresis (LCP) at University Hospital Olomouc. Clinical data were retrospectively collected from medical records. The primary objective was to evaluate the procedural efficacy and safety. Clinical and laboratory data were analyzed. Survival outcomes were assessed by Kaplan–Meier analysis and compared using the log‐rank test. Median age at diagnosis was 57 years. Dyspnea (60.0%), neuropsychiatric symptoms (31.7%), and visual impairment (6.2%) were the most common leukostasis manifestations. LCP effectively reduced WBC counts without significant adverse events, median of 2.2 TBV was treated, and 52.3% of the patients requiring more than one session. *FLT3*‐ITD and *NPM1* mutations were detected in 26/46 (56.5%) and 17/43 (39.5%), respectively, *KMT2A* rearrangements were present in 5/57 (8.8%). Intensive chemotherapy was feasible in 56.9% of patients, with 26.2% undergoing allo‐HSCT. Median OS was 5.9 months (95% CI: 1.3–8.4), significantly longer in therapy‐eligible patients, but outcomes remained poor, highlighting HL as an unmet clinical need. LCP remains a valuable therapeutic option for patients with HL in newly diagnosed AML. Our long‐term experience supports its safety and efficacy, particularly in symptomatic patients, as a bridge to definitive therapy regardless of treatment intensity eligibility.

## 1 Introduction

Acute myeloid leukemia (AML) is a genetically heterogeneous clonal disorder of hematopoiesis, with an incidence of 3–4 cases per 100 000 people and a median age at diagnosis of 68 years [1]. In newly diagnosed AML, approximately 5%–20% of patients present with hyperleukocytosis [1, 2].

Hyperleukocytosis (HL) is usually defined as a white blood cell (WBC) count exceeding 100 000/μL, resulting from the uncontrolled proliferation of leukemic cells [3]. It is not the elevated WBC count itself that poses the primary danger, but rather the associated complications, such as leukostasis, tumor lysis syndrome (TLS), and disseminated intravascular coagulation (DIC), that put patients at serious risk and require immediate treatment. Notably, clinical symptoms may occur even at lower WBC counts [4].

HL is more frequent and severe in acute leukemias compared to chronic ones, with leukostasis being particularly common in AML due to several contributing factors [5]. HL is associated with dismal prognosis due to a greater likelihood of early death caused by HL‐related complications, an increased risk of relapse and mortality over time. Therefore, HL is a hematologic emergency, demanding prompt intervention to reduce the risk of early death [2, 6, 7].

HL in AML is associated with certain features, including monocytic subtypes, FMS‐like tyrosine kinase 3 (*FLT3*), nucleophosmin 1 (*NPM1*), CCAAT/enhancer binding protein alpha (*CEBPA*), DNA (cytosine‐5)‐methyltransferase 3A (*DNMT3A*) and Tet methylcytosine dioxygenase 2 (*TET2*) gene mutations, chromosomal aberration involving histone‐lysine *N*‐methyltransferase 2A (*KMT2A*) and core‐binding factor beta (*CBFB*) gene, and elevated lactate dehydrogenase (LDH) [2, 3, 8, 9, 10, 11, 12, 13].

Two main mechanisms underline HL: rapid proliferation of leukemic blasts and disrupted adhesion of hematopoietic cells, reducing their retention in the bone marrow [14, 15, 16].

Leukostasis results from both mechanical and cellular factors. Myeloid blasts are large and less deformable, contributing to microvascular obstruction. Additionally, leukemic blasts interact with endothelial cells via upregulated adhesion molecules (e.g., ICAM‐1, VCAM‐1, E‐selectin), enhancing endothelial binding and inflammation [17, 18, 19]. Cytokine release, such as TNF‐α and IL‐1β, promotes this process and may cause endothelial damage, hemorrhage, and tissue injury [2].

Early mortality is significantly higher in patients with HL, up to 20% in the first week, compared to 3%–9% in AML without HL. Main causes of early death include bleeding, thromboembolic events, and neurologic or pulmonary complications. HL also negatively affects long‐term outcomes, with lower overall survival, even when accounting for other prognostic factors [6, 13, 20].

### 1.1 Leukostasis and Its Management

Leukostasis usually presents with respiratory and neurologic symptoms and affects about 44%–50% of AML patients with WBC > 100 000/μL. Organs most commonly involved are lungs, brain, and kidneys. Symptoms can mimic infection or hemorrhage, making diagnosis challenging. Leukostasis contributes significantly to early death and requires rapid initiation of cytoreductive therapy [3, 6, 21, 22].

Hydroxyurea (HU) is often used to lower leukocyte counts before intensive chemotherapy (7 + 3‐based regimen) or before hypomethylating agent (HMA) based regimen. In acute promyelocytic leukemia, all‐trans retinoic acid (ATRA) should be started immediately if suspected, however, initiation may trigger leukocytosis due to differentiation syndrome [21, 22].

Therapeutic leukocytapheresis (LCP) mechanically reduces WBC count and is available in many treatment centers. When performed by experienced healthcare professionals, it is generally safe and effective. Currently, LCP is indicated only for patients with clinical signs of leukostasis [1, 3, 7, 23, 24, 25, 26, 27]. Careful monitoring during the procedure is essential, particularly fluid balance and vital signs. The risks of hypocalcemia and bleeding are manageable with calcium supplementation, by adjusting the dose of acid‐citrate dextrose solution A (ACD‐A) and by coagulation factor and platelet substitution [21, 22].

## 2 Materials and Methods

### 2.1 Patient Cohort

From November 1st 2005 to September 30th 2025 total of 69 patients with AML underwent LCP at the Department of Hemato‐Oncology, University Hospital Olomouc, Czech Republic. Four patients presented with HL at the time of relapse and were excluded from the study. Data from all patients, obtained from available medical records, were processed anonymously in accordance with the Code of Ethics of Olomouc University Hospital and the Declaration of Helsinki.

The procedure was performed using either the Cobe Spectra or Spectra Optia Apheresis System (both Terumo BCT, Lakewood, CO, USA) with use of ACD‐A and intravenous calcium supplementation. No sedimenting agent (e.g., hydroxyethyl starch) was used. The decision whether to perform LCP was made based on clinical judgment, taking into account the patient's symptoms and performance status as classified by the Eastern Cooperative Oncology Group (ECOG). Patients with WBC < 100 000/μL were included if they presented with clinical symptoms consistent with leukostasis. The primary objective was to evaluate the procedural efficacy and safety. Secondary exploratory objectives included the assessment of clinical outcomes and leukostasis‐related symptom relief. However, due to the retrospective design of this study, clinical response criteria and time to symptom improvement after LCP were not predefined or systematically recorded. As a result, quantitative assessment of post‐procedure improvement in leukostasis‐related symptoms could not be consistently performed. Cytoreduction with HU was initiated no later than the day LCP was started, during which the administration of corticosteroids (e.g., dexamethasone) was at the physician's discretion. Crystalloids were used for fluid replacement. The bloodstream was accessed via a peripheral or central venous catheter.

### 2.2 Immunophenotyping

The immunophenotyping was performed by multiparameter flow cytometry successively on FACS Calibur (CellQuest Pro Software, Becton Dickinson), FACS Canto II (FACS Diva, Becton Dickinson) and Omnicyte (Infinicyte, Cytognos). Flow cytometry was performed on blast cells gated on their abnormal light scatter characteristics. Standard panel of analyzed antigens included CD10, CD19, CD20, CD22, CD24, CD2, CD3, CD5, CD7, CD4, CD8, CD34, CD33, CD13, CD14. CD15, CD65, HLA‐DR, CD117, CD79a, TdT, and MPO. If necessary, the cells were labeled with antibodies to CD11c, CD41, CD61, CD64, CD71, and CD235a. A membrane marker was considered positive when more than 20% of the blast cells expressed it. A cytoplasmic marker was considered positive when more than 20% of the blast cells expressed it.

### 2.3 Cytogenomic and Molecular Analysis

Leukemic cells were obtained from the bone marrow (BM) or peripheral blood (PB) at the time of diagnosis. Before analysis, cells were cultured for 24 h in RPMI 1640 medium or BM medium (Gibco, USA; Biological Industries, USA). Banding of chromosomes was done using standard G‐banding procedures. Chromosome analysis was performed using the Olympus BX60 microscope and Ikaros software (MetaSystems, Germany), at least 20 metaphases and 300 interphase cells were evaluated for each patient. The karyotypes were compiled according to the ISCN 2024 [28]. Patients were examined by fluorescence in situ hybridization (FISH) with a commercially available XL probes (MetaSystems): *MECOM* 3q26, Del(5)(q31), Del(7)(q22q31), t(8;21) plus, KMT2A BA, ETV6, CBFB.

DNA and RNA for molecular analyses were extracted from BM or PB at the time of diagnosis. Gene fusions/rearrangements (*KMT2A*, *RUNX1T1::RUNX1*, *CBFB::MYH11*, *PML::RARA*) were detected by nested PCR (van Dongen et al. 1999), gene mutations (*NPM1* Fallini et al. 2009, *FLT3*‐ITD/TKD, *cKIT* Schumacher et al. 2008, *CEBPA* Tien et al. 2005, *IDH1*/*2*, and others) by fragmental analysis, Sanger sequencing and MPS (Massively parallel sequencing). For library preparation, SureSelect XT HS2 kit (Agilent) with molecular barcodes were used. Sequencing were performed on Miseq and NextSeq 2000 with pair end reading. Detected variants were classified using NCBI SNP database, Clinvar and Varsome.

### 2.4 Statistical Analysis

All statistical analyses were performed using IBM SPSS Statistics, version 23 (Armonk, NY: IBM Corp.). Quantitative variables were compared with the Mann–Whitney *U* test, and qualitative variables with the chi‐square or Fisher's exact test. Data normality was assessed using the Shapiro–Wilk test. Overall survival (OS) was estimated by the Kaplan–Meier method and compared using the log‐rank test. A *p*‐value < 0.05 was considered statistically significant.

## 3 Results

### 3.1 Clinical Characteristics

A total of 65 patients were included in the analysis, comprising 29 females (44.6%) and 36 males (55.4%), with a median age of 57 years (range 19–78). Table 1 Most common clinical symptoms of leukostasis were dyspnea (*n* = 39; 60.0%), neuropsychiatric disorders (i.e., confusion, dizziness, headache, impaired consciousness, or epileptic seizures) (*n* = 20; 30.8%) and vision impairment with/without confirmed retinal hemorrhage (*n* = 4; 6.2%). In all patients with neurological symptoms, brain computed tomography (CT) was performed to exclude intracranial hemorrhage. Imaging was conducted either at our center or at referring hospitals prior to transfer. Patients presenting with seizures or moderate to severe impairment of consciousness (Glasgow Coma Scale < 13) were assessed by a neurologist, with recommendations for appropriate treatment and diagnostic workup. Clinical presentations were often cumulative, 15 (23.1%) patients had at least two symptoms. One patient (1.5%) required cardiopulmonary resuscitation due to leukostasis, which resulted in respiratory insufficiency with secondary myocardial ischemia. Six patients (9.2%) were asymptomatic, in these cases, LCP was considered prophylactic. No significant difference in age was observed between asymptomatic and symptomatic patients (median age, 50 years vs. 57 years, *p* = 0.440).

**TABLE 1:** Baseline characteristics.

Characteristic	All patients (*n* = 65)	Intensive therapy eligible (*n* = 37)	Intensive therapy non‐eligible (*n* = 28)	*p*
Sex				
Male—no. (%)	36 (55.4)	20 (54.1)	16 (57.1)	0.840
Female—no. (%)	29 (44.6)	17 (45.9)	12 (42.9)	
Median age at the diagnosis (range)	57 (19–78)	47 (20–65)	68 (19–78)	< 0.0001
ECOG				
0–1—no. (%)	25 (38.5)	17 (45.9)	8 (28.6)	0.154
≥ 2—no. (%)	40 (61.5)	20 (54.1)	20 (71.4)	
Median baseline parameters prior first leukocytapheresis				
White blood cells [× 10^9/L] (range)	178 (40–434)	163 (67–381)	222 (40–434)	0.177
Hemoglobin [g/L] (range)	86 (29–141)	86 (29–129)	87 (59–141)	0.633
Hematocrit [%] (range)	26 (10–41)	26 (10–41)	27 (18–40)	0.825
Platelets [× 10^9/L] (range)	44 (11–204)	40 (11–144)	47 (18–204)	0.315
Leukocytapheresis parameters				
Median TBV at first LCP [mL] (range)	5246 (3204–7757)	5403 (3204–7757)	5118 (3323–6648)	0.839
Median of treated TBV at first LCP (range)	2.2 (0.6–3.2)	2.3 (0.6–3.2)	2.1 (0.9–3.1)	0.092
Need for ≥ 2 leukocytapheresis—no. (%)	34 (52.3)	19 (51.4)	15 (53.6)	0.859
*NPM1* mutation status (data were available for 43 patients, 26 in the eligible group, and 17 in the non‐eligible group)				
Mutated—no. (%)	17 (39.5)	11 (42.3)	6 (21.4)	0.646
Wild‐type—no. (%)	26 (60.5)	15 (57.7)	11 (39.3)	
*FLT3*‐ITD mutation status (data were available for 46 patients, 29 in the eligible group, and 17 in the non‐eligible group)				
Mutated—no. (%)	26 (56.5)	16 (55.2)	10 (58.8)	0.810
Wild‐type—no. (%)	20 (43.5)	13 (44.8)	7 (41.2)	
Cytogenomic changes (data were available for 57 patients, 36 in the eligible group, and 21 in the non‐eligible group)				
Normal karyotype—no. (%)	31 (54.4)	21 (58.3)	10 (47.6)	0.433
Aberrant karyotype (included complex)—no. (%)	26 (45.6)	15 (41.7)	11 (52.4)	
Complex karyotype—no. (%)	3 (5.3)	0	3 (14.3)	0.020
inv(16)—no. (%)	5 (8.8)	5 (13.9)	0	0.074
*KMT2A* rearrangement—no. (%)	5 (8.8)	3 (8.3)	2 (9.5)	0.878
t(9;22)—no. (%)	1 (1.8)	0	1 (4.8)	NA
t(14;15)—no. (%)	1 (1.8)	1 (2.8)	0	NA
Other adverse risk changes (monosomy, del5q, del7q, *MECOM* rearrangement)—no. (%)	6 (10.5)	1 (2.8)	5 (23.8)	0.013
European leukemia network 2022				
Favorable risk—no. (%)	6 (9.2)	5 (13.5)	1 (3.6)	0.170
Intermediate risk—no. (%)	35 (53.8)	25 (67.6)	10 (35.7)	0.011
Adverse risk—no. (%)	17 (26.2)	7 (18.9)	10 (35.7)	0.127
Unknown—no. (%)	7 (10.8)	0	7 (25.0)	0.001
Therapy‐related—no. (%)	6 (9.2)	3 (8.1)	3 (10.7)	0.719

Abbreviations: ECOG: Eastern Cooperative Oncology Group, *FLT3*: FMS‐like tyrosine kinase 3, inv: inversion, ITD: internal tandem duplication, *KMT2A*: histone‐lysine N‐methyltransferase 2A, *MECOM*: MDS1 and EVI1 complex locus protein, NA: not assessed, *NPM1*: nukleophosmin, TBV: total blood volume.

### 3.2 Laboratory and Apheresis Parameters

The median WBC prior to LCP was 176 000/μL (range 44 000–434 000), which decreased to a median of 73 000/μL (range 21 000–360 000) following the first procedure. Baseline parameters included a median hemoglobin level of 86 g/L (range 29–141), hematocrit of 0.26 (range 0.10–0.41), platelet count of 44 000/μL (range 11 000–204 000), fibrinogen level of 3.10 g/L (range 0.53–8.18), and estimated glomerular filtration rate (eGFR) of 0.96 mL/s/1.73 m^2^ (range 0.12–1.5). Elevated lactate dehydrogenase (LDH) level, defined as more than twice the upper limit of normal (≥ 1200.0 U/L), was observed in 33 patients (50.8%). The median total blood volume (TBV) was 5246 mL (range 3204–7757), and a median of 2.2 TBV (range 0.6–3.2) was treated.

In prophylactic subgroup the median WBC prior to LCP was slightly higher compared to the rest of the studied population (median WBC, 192 000/μL vs. 176 000/μL, *p* = 0.510), which decreased to a median of 94 000/μL (range 39 000–196 000) following the prophylactic LCP.

A second LCP on the consecutive day was required in 34 patients (52.3%), while 3 (4.6%) patients underwent three consecutive sessions. During the second procedure, the median WBC prior to the procedure was 106 000/μL (range 45 000–323 000), which decreased to a median of 46 000/μL (range 19 000–209 000) following cell separation. The median hemoglobin level was 90 g/L (range 60–121), hematocrit of 0.26 (range 0.18–0.36), platelet count 37 000/μL (range 18 000–166 000), fibrinogen level 2.6 g/L (range 0.99–4.70), and the estimated glomerular filtration rate (eGFR) was 1.14 mL/s/1.73 m^2^ (range 0.2–1.5). At the second LCP, the median TBV was 5511 mL (range 3654–7457), and a median of 2.2. TBV (range 0.3–3.0) was treated.

All adverse events observed during apheresis were related to intravenous access (e.g., malposition, dysfunction, local bleeding).

### 3.3 Immunophenotypic Features

Based on flowcytometric analysis, at least one progenitor marker (i.e., CD34, CD117, and/or HLA‐DR) was identified in 57 cases (87.7%), while markers associated with monocytic differentiation (i.e., CD11c, CD14, and/or CD64) were positive in 50 samples (76.9%). The majority of monocytic features correlated with morphological findings, which were most commonly classified as M4 or M5 (myelomonocytic or monocytic) according to the French‐American‐British (FAB) classification. Additionally, five patients (7.7%) harbored an APL‐like immunophenotype (i.e., CD34 and HLA‐DR negative; CD117 and myeloperoxidase strongly positive) [29]. In two cases (3.1%) the immunophenotype is unknown.

### 3.4 Cytogenomic and Molecular Findings

In terms of cytogenomic findings, 31 patients (47.7%) exhibited a normal karyotype, 26 patients (40.0%) harbored structural or numerical abnormalities (e.g., translocation, deletion, aneuploidy, or complex changes), and in 8 patients (12.3%) the karyotype could not be determined. Table 1 The most frequent structural abnormalities were inv(16)(p13.1q22) and *KMT2A* (formerly *MLL*) rearrangements, each detected in 5/57 tested patients (8.8%). Two patients with inv(16) were also positive for a *C‐KIT* mutation. The spectrum of *KMT2A* rearrangements included t(6;11)(q27;q23) (*KMT2A::MLLT4*), t(9;11)(p22;q23) (*KMT2A::MLLT3*), t(10;11)(p12;q23) (*KMT2A::MLLT10*), and t(11;19)(q23;p13.1) (*KMT2A::ELL*). A complex karyotype, defined as the presence of at least three clonal cytogenomic abnormalities, was identified in three patients (4.6%). Additionally, t(15;17)(q24.1;q21.2) (*PML::RARA*) and t(9;22)(q34;q11) (*BCR::ABL*) were each observed in a single patient.

With respect to available molecular data, *FLT3*‐ITD was present in 26/46 tested patients (56.5%), and *NPM1* mutation was detected in 17/43 tested patients (39.5%), with concurrent occurrence in 11 patients. *FLT3* and *NPM1* mutation status was unknown in 19/65 (29.2%) and 22/65 (33.8%) patients, respectively. Additionally, an NGS panel for selected gene mutations was performed in eight patients, identifying HL‐related mutations in *DNMT3A* and *TET2* in four and one cases, respectively. Based on available cytogenomic and molecular findings, risk stratification according to the 2022 European Leukemia Network (ELN22) classification identified 6 patients (9.2%) as favorable risk (FR), 35 patients (53.8%) as intermediate risk (IR), and 17 patients (26.2%) as adverse risk (AR), in 7 patients (10.8%), risk categorization was not feasible (Table 1).

### 3.5 Treatment‐Eligibility and High Rate of Salvage Need

**FIGURE 1:**
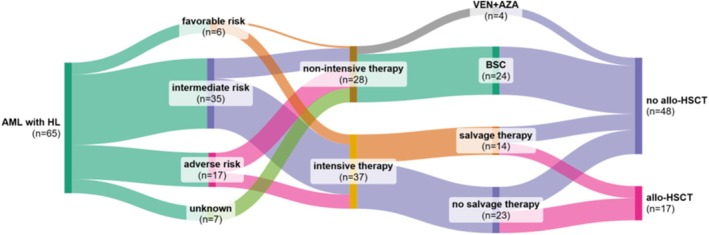
Sankey diagram showing patient flow across treatment groups according to ELN 2022 risk stratification. Abbreviations: allo‐HSCT, allogeneic hematopoietic stem cell transplantation; AML with HL, acute myeloid leukemia with hyperleukocytosis; BSC, best supportive care (cytoreduction, transfusion, anti‐infective drugs, and symptom alleviation); VEN+AZA, venetoclax plus azacitidine.

Intensive induction chemotherapy was initiated in 37 patients (56.9%) who were deemed eligible based on performance status and comorbidities. Intensively treated patients were significantly younger compared with those who were not eligible (median age, 47 vs. 68 years; *p* < 0.0001). Table 1 The standard 7 + 3‐based regimen was given with or without the addition of gemtuzumab ozogamicin (GO) and/or a FLT3 inhibitor (e.g., midostaurin) depending on molecular profile and clinical judgment. The induction therapy was followed by consolidation cytarabine‐based therapy. In the case of APL, the treatment strategy consisted of a combination of idarubicin and ATRA. The incorporation of targeted therapies was largely influenced by reimbursement policies and drug availability at the time of treatment initiation. Out of the treated group, 14 patients (37.8%) required salvage therapy due to refractory disease or early relapse. The most commonly used regimens were FLAG (fludarabine, cytarabine, G‐CSF) with/without idarubicin, and MIDAM+GO (mitoxantrone, idarubicin, cytarabine with GO). Allogeneic hematopoetic stem cell transplantation (allo‐HSCT) was performed in 17 patients (45.9%). Figure 1 In addition, autologous HSCT was performed in three patients with AML and inv(16), as this used to be the standard of care at that time.

In 24 patients (36.9%), a decision was made to proceed with best supportive care (BSC), which included cytoreduction using HU or low‐dose cytarabine as clinically indicated. In the case of Philadelphia chromose‐positive disease, treatment included a tyrosine kinase inhibitor dasatinib. Additionally, four patients received the venetoclax plus azacitidine (VEN+AZA) regimen, which was not intended as a bridge to allo‐HSCT (Figure 1).

### 3.6 Hyperleukocytosis Remains Unmet Need

**FIGURE 2:**
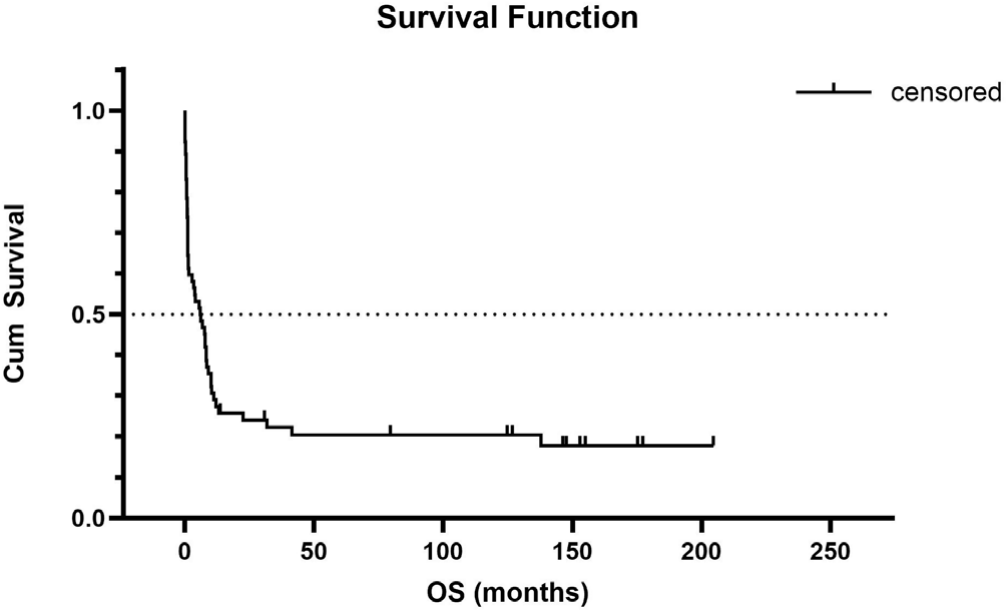
Kaplan–Meier curve of overall survival (OS) in AML patients with hyperleukocytosis after leukocytapheresis. Median OS: 5.9 months (95% CI: 1.3–8.4).

**FIGURE 3:**
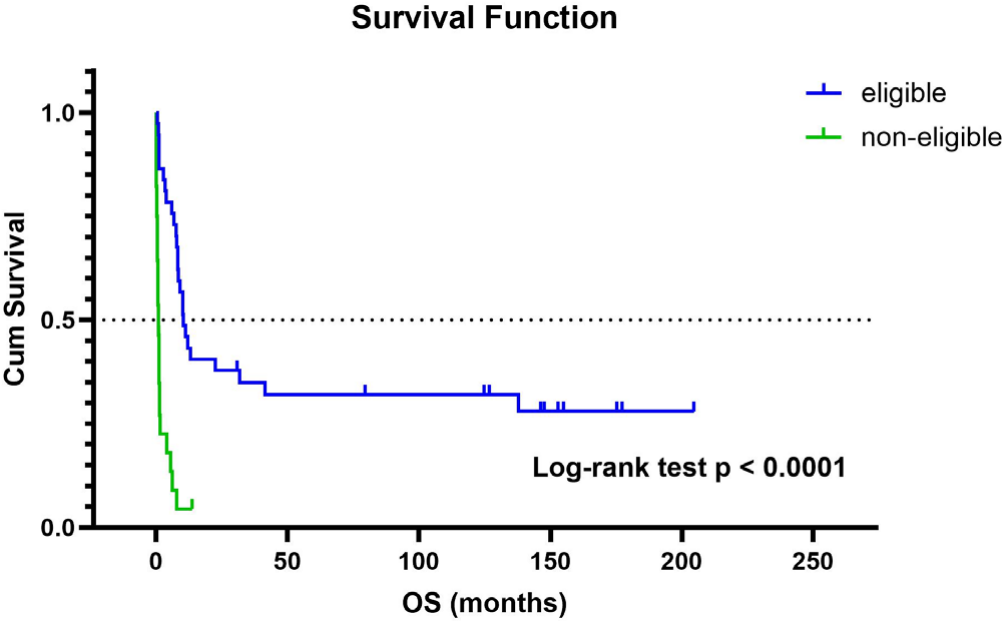
Kaplan–Meier curves of overall survival (OS) in patients eligible for intensive therapy (blue) and those not eligible (green). Median OS: 10.3 months (95% CI: 8.2–41.5) versus 0.95 months (95% CI: 0.6–1.3); log‐rank test *p* < 0.0001.

Survival analysis was performed to evaluate overall survival (OS) in relation to treatment eligibility and ELN22 risk groups. The median OS for the entire cohort was 5.9 months (95% CI: 1.3–8.4). There was no significant difference in the median OS of patients with ≥ 2 leukostasis‐related symptoms and those with only one symptom (5.9 months [95% CI: 0.1–10.1] vs. 6.2 months [95% CI: 1.2–8.4], log‐rank test *p* = 0.392). As well as, there was no significant difference in the median OS of patients with WBC ≥ 100 000/μL and those with < 100 000/μL (8.3 months [95% CI: 0.6–not reached] vs. 5.6 months [95% CI: 1.2–8.4], log‐rank test *p* = 0.346). The estimated 3‐months and 1‐year survival rates were 58.1% (95% CI: 45.1%–69.0%) and 29.0% (95% CI: 17.5%–39.6%), respectively (Figure 2). As expected, patients eligible for therapy had a significantly longer median OS compared with those who were not eligible (10.3 months [95% CI: 8.2–41.5] vs. 0.95 months [95% CI: 0.6–1.3], log‐rank test *p* < 0,0001). Seven patients (10.8%) and 17 patients (26.2%) died within 7 days and 30 days after LCP, respectively. Causes of early death included AML progression, septic shock with multiple organ dysfunction syndrome (MODS), and/or severe hemorrhage. Among intensively treated patients, the estimated 3‐months and 1‐year survival rates were 83.8% (95% CI: 71.9%–95.7%) and 45.9% (95% CI: 29.9%–62.0%), respectively (Figure 3).

**FIGURE 4:**
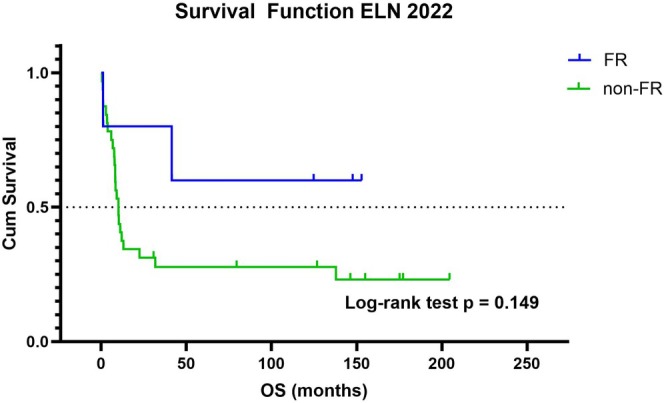
Kaplan–Meier curves of overall survival (OS) in the favorable‐risk group (FR, blue) and the non‐favorable‐risk group (non‐FR, green). Median OS: Not reached versus 10.2 months (95% CI: 7.7–22.5); log‐rank test *p* = 0.149.

Given that the ELN22 risk stratification was primarily developed for patients receiving intensive treatment, we further analyzed survival data within the intensive therapy‐eligible group. No significant difference in median OS rate was observed between FR and non‐FR (IR and AR) risk group, mOS: not reached versus 10.2 months (95% CI: 7.7–22.5); log‐rank test *p* = 0.14 (Figure 4). The median follow‐up after allo‐HSCT was 27.4 months (range 2.8–193.0). Post‐transplant relapse occurred in 35% of patients (*n* = 6), with a median time to relapse of 2.7 months (range 1.9–4.4). All relapsed patients died within 4.5 months.

## 4 Discussion

Despite the fact that much recent work has extensively characterized the genetic alterations in AML with respect to prognostic classification and potential therapeutic targets, HL still represents an adverse condition.

LCP may be considered as an adjunctive measure in patients with symptomatic leukostasis to facilitate rapid cytoreduction and short‐term clinical stabilization. According to the current American Society for Apheresis (ASFA) 2023 guidelines, LCP for symptomatic leukostasis is classified as a Category III indication with a Grade 2B level of evidence, reflecting limited and mixed data regarding its impact on survival. Firstly, we show that LCP is a safe and potentially life‐saving procedure that may help relieve symptoms of leukostasis and serve as a bridge to treatment decisions. The procedure should be performed in accordance with the current state of knowledge and is recommended only for symptomatic patients [1, 3, 7, 20, 23, 24, 25, 26]. Currently, the treated volume should be between 1.5 and 2 TBV, as recommended by ASFA [27].

Although LCP is primarily intended to relieve leukostasis‐related symptoms, the impact on specific clinical manifestations and time to improvement could not be reliably quantified in this retrospective cohort, underscoring the need for prospective studies with standardized response criteria.

The results of the present study do not allow conclusions regarding a survival benefit of LCP in patients who are non‐eligible for intensive treatment. However, LCP may still represent a clinically relevant supportive intervention. In this setting, LCP should be considered primarily for rapid cytoreduction, symptomatic relief, and potential improvement in quality‐of‐life rather than survival prolongation. This approach is supported by the availability of less intensive treatment options (e.g., VEN+AZA), and is consistent with the goals of palliative care, where symptom control and quality‐of‐life represent key therapeutic objectives [30].

An association between several AML characteristics and HL has been described. In this study, more than a half of analyzed samples was positive for *FLT3*‐ITD (26/46; 56.5%), *NPM1* mutations were detected in 17/43 analyzed samples (39.5%), and four distinct *KMT2A* rearrangements were identified in 5/57 analyzed samples (8.8%). The incidence of *FLT3*‐ITD and *KMT2A* rearrangements was higher than that reported in the general AML population (approximately 30% for *FLT3*‐ITD, and 2% for *KMT2A* rearrangements) [3, 31, 32]. In contrast, *NPM1* mutations were observed at a frequency consistent with previous reports (approximately 30% overall, and 40%–60% in AML with normal karyotype) [3, 31, 32]. Identifying mentioned alterations is crucial for decisions on targeted therapy (e.g., midostaurin, gilteritinib, menin inhibitors) and for risk stratification with respect to guiding the patient toward allo‐HSCT. Compared with large real‐world cohorts, our study shows a markedly lower proportion of favorable risk patients and a higher proportion of intermediate risk cases. The relatively large unclassified fraction further limits direct comparability [33].

To date, data on targeted therapy in patients with HL remain very limited, as these patients are usually underrepresented or even excluded from clinical trials. Subgroup analyses specifically for HL are not available, including in pivotal studies such as RATIFY and ADMIRAL [34, 35]. Importantly, except for the RATIFY trial, most published studies have not been conducted in newly diagnosed disease. Consequently, the interpretation of clinical trial results in HL patients is challenging and underscores the need for dedicated studies or at least subgroup analyses. Most current knowledge on the efficacy of novel agents in HL comes from case reports or small pediatric cohorts, which highlights the limitations of current evidence in HL patients. For example, sorafenib (a *FLT3* inhibitor) has been successfully used as a cytoreductive agent in HL in pediatric *FLT3*‐ITD mutated AML [36]. Moreover, dexamethasone administration has been established as standard of care at our center, reflecting its well‐documented clinical benefit [37]. In contrast, the use of *IDH1/2* or menin inhibitors is more challenging in HL setting, as these agents may exacerbate leukocytosis due to differentiation syndrome [38, 39, 40]. Grade 3 or higher leukocytosis has been reported in 1.7%–6% of patients treated with *IDH1/2* inhibitor [38, 39]. A further HL‐associated feature, a monocytic‐like immunophenotype, was identified in nearly 80% samples. Such AML cases are often resistant to venetoclax‐based regimens [41]. Subgroup analyses of novel treatment efficacy in monocytic‐differentiated AML are therefore warranted.

As mentioned, HL is an independent poor prognostic factor irrespective of cytogenomic and mutation status, this finding is further corroborated by our results. Achieving and maintaining a first complete remission (CR) is crucial in AML patients, but treatment may fail due to relapse from CR, primary induction failure or treatment‐related mortality. As shown in our study, almost 60% of patients underwent intensive therapy, of these, more than one third required salvage treatment due to refractory disease or early relapse. Our findings are consistent with population‐based and real‐world data, where 36%–58% of adults receive intensive chemotherapy depending on age, performance status, and comorbidities [42, 43, 44]. Among intensively treated patients, primary refractory AML occurs in about 10%–30%, and the annual relapse risk during the first year after CR approaches 40%, indicating that more than one third typically require salvage therapy for refractory disease or early relapse [45, 46, 47, 48]. Allo‐HSCT plays an important and complex role in post‐remission treatment due to its strong graft‐versus‐leukemia (GvL) effect [49, 50]. However, in patients with favorable risk disease, the indication and timing of allo‐HSCT are currently guided primarily by minimal residual disease (MRD) status. In our cohort, allo‐HSCT was performed in nearly half of intensive therapy eligible patients, and it seems to partially amelioratethe the poor prognostic impact of HL. Relapse after allo‐HSCT is associated with a dismal prognosis [51, 52]. Notably, our small cohort had an even worse outcome compared with previously reported data [52].

## 5 Conclusion

In this study, we report our long‐term experience with LCP in newly diagnosed AML. Our findings confirm that LCP represents an effective management strategy for HL, and indicate potential benefits in symptomatic patients, both those eligible and ineligible for intensive therapy. It may serve as a bridge‐to‐decision in the current era of less‐intensive regimens, however, its impact on survival remains unclear. Nevertheless, interpretation of these results is limited by the relatively small cohort. HL in AML remains a major clinical challenge and an unmet need. Further prospective, multicenter studies are warranted to optimize its management in the context of emerging therapeutic approaches.

## Author Contributions

V.L. drafted the original manuscript. V.L. and I.S. designed the study and collected the data. M.P., T.S., M.C., L.R., and E.F. treated the patients. J.N. and H.U. analyzed the cytogenomic and molecular data. Z.P. performed the flow cytometric assessments. E.F. and T.P. supervised the study. All authors critically revised the manuscript and approved the final version.

## Funding

This work was supported by Univerzita Palackého v Olomouci (IGA_LF_2025_005) and Ministerstvo Zdravotnictví Ceské Republiky (FNOl, 00098892).

## Ethics Statement

Ethics committee review and approval were waived for this study, as all healthcare provided was part of standard diagnostic and therapeutic procedures, approval from the Ethics Committee University Hospital Olomouc was therefore not required.

## Consent

Patient consent was waived for this study, as all procedures were performed as part of routine diagnostic and therapeutic care, and no identifiable patient data are included.

## Conflicts of Interest

The authors declare no conflicts of interest.

## Data Availability

All data supporting the findings of this study are available within the paper.
